# Computational Identification of Milk Trait Regulation Through Transcription Factor Cooperation in Murciano-Granadina Goats

**DOI:** 10.3390/biology13110929

**Published:** 2024-11-15

**Authors:** Muhammad Imran Khan, Hendrik Bertram, Armin Otto Schmitt, Faisal Ramzan, Mehmet Gültas

**Affiliations:** 1 Faculty of Agriculture, South Westphalia University of Applied Sciences, Lübecker Ring 2, 59494 Soest, Germany; bertram.hendrik@fh-swf.de; 2 Breeding Informatics Group, Department of Animal Sciences, Georg-August University, Margarethe von Wrangell-Weg 7, 37075 Göttingen, Germany; armin.schmitt@uni-goettingen.de; 3 Department of Livestock Production and Management, Faculty of Veterinary and Animal Sciences, PMAS-Arid Agriculture University, Rawalpindi 46300, Pakistan; 4 Center for Integrated Breeding Research (CiBreed), Georg-August University, Carl-Sprengel-Weg 1, 37075 Göttingen, Germany; 5 Institute of Animal and Dairy Sciences, Faculty of Animal Husbandry, University of Agriculture, Faisalabad 38000, Pakistan; drfaisal.ramzan@uaf.edu.pk

**Keywords:** Murciano-Granadina, milk traits, random forest, transcription factors, mammary gland

## Abstract

The Murciano-Granadina goat (MUG) is a renowned dairy breed, known for its adaptability and resilience, as well as for its exceptional milk traits characterized by high protein and fat content, along with low somatic cell counts. These traits are governed by complex biological processes, crucial in shaping phenotypic diversity. Thus, it is imperative to explore the factors regulating milk production and lactation for this breed. In this study, we investigated the genetic architecture of seven milk traits in MUGs, employing a two-step computational analysis to examine genotype–phenotype associations. Initially, a random forest algorithm identified the relative importance of each single-nucleotide polymorphism (SNP) in determining the traits of interest. The second step applied an information theory-based approach to exploring the complex genetic architecture of quantitative milk traits, focusing on epistatic interactions that may have been overlooked in the first step. These approaches allowed us to identify an almost distinct set of candidate genes for each trait. In contrast, by analyzing the promoter regions of these genes, we revealed common regulatory networks among the milk traits under study. These findings are crucial for understanding the molecular mechanisms underlying gene regulation, and they highlight the pivotal role of transcription factors (TFs) and their preferential interactions in the development of these traits. Notably, TFs such as DBP, HAND1E47, HOXA4, PPARA, and THAP1 were consistently identified for all traits, highlighting their important roles in immunity within the mammary gland and milk production during lactation.

## Simple Summary

Milk production and its composition are important for the economy, and they depend on complex biological processes. By finding the key genes and causal mutations linked to milk yield, we can improve breeding strategies for dairy animals. Thanks to advanced bioinformatics tools, it is now easier to find the genetic factors that affect milk traits. In our study, we used these methods to explore the genetics of milk traits in Murciano-Granadina goats. Although we found distinct genes associated with each trait, the regulatory proteins showed shared-yet-dynamic roles in controlling gene activity across different traits. This helped us understand how genes work together in the mammary gland, which affects milk production and udder health.

## 1. Introduction

The Murciano-Granadina goat (MUG), a prominent dairy breed from Spain, is closely associated with the regions of Murcia and Granada, reflecting its historical origins. This breed has been introduced to various farming systems, potentially even beyond its native region, due to its resilience in semi-arid conditions and its ability to cope with fluctuating food availability [1]. Morphologically, MUGs typically have a medium build, weighing around 50 kg, and brown or black coats. They may either be horned or hornless. Furthermore, this breed consistently exhibits a polyestrous nature, with notable prolificacy, often giving birth to two or more kids and demonstrating longevity with up to six births [2,3]. To improve the morphology, milk production, and milk composition of MUGs, the National Association of Murciano-Granadina Goat Breeders (CAPRIGRAN) started a breeding program in 2012. As a result, the MUG can now produce an average of 584 kg of milk during the 287 days of lactation cycle, with average fat, protein, and lactose contents of 5.3, 3.6, and 4.7 percent, respectively [1]. The main use of goat milk in Spain is to manufacture cheese, and the MUG breed is the best choice for this purpose because of its high protein and fat content and its low somatic cell counts [4,5,6].

Several association studies have pinpointed genomic regions and the distribution of single-nucleotide polymorphisms (SNPs) affecting caprine milk production and composition [7,8,9,10,11,12,13]. Similarly, for the MUG breed, identifying the genetic variants impacting milk traits has been the subject of numerous studies [6,14,15,16,17,18,19,20,21,22,23,24,25], as presented in Table 1.

In particular, these studies explored various genetic and molecular factors in MUGs, including the effects on milk quality, the impact of casein complex haplotype variants on production and composition, the relationship between genetic variations and dairy traits, and the use of genetic markers from artificial selection in milk trait associations. Predominantly, these studies focused on the additive effects of genotypes, analyzing associations between single SNPs and traits of interest. However, it is well known that epistasis among genes plays a critical role in shaping the complex inheritance mechanisms of quantitative traits, an aspect that single-SNP-based association studies may miss [26]. Consequently, a range of computational tools has been developed to detect and incorporate epistasis into analytical frameworks, thereby enhancing the efficacy of single-SNP models [27,28,29,30]. These tools improve predictive capabilities in association analysis and help decipher the complex genetic mechanisms underlying quantitative traits [27,31]. In this context, Liang et al. [32] demonstrated that including both additive and epistatic SNP effects significantly improved the accuracy of predicting residual feed intake in US Holstein cows. Furthermore, Prakapenka et al. [33] extended their initial single-SNP model [34] by emphasizing the role of epistasis in dairy production and fertility traits among first-lactation US Holstein cattle. Similarly, Pizarro et al. [18] showed in their study on MUGs that incorporating epistasis effects, particularly of SNPs related to the casein complex, resulted in better predictive accuracy for breeding-value estimations of dairy traits compared to models that only considered additive effects.

Complementing these genetic studies, another crucial aspect in unraveling the complex genetic architecture of milk-related traits involves investigating their transcriptional gene regulatory mechanisms. Although these mechanisms are often understudied, they are essential for controlling the biological processes underlying quantitative traits in several organisms [35,36]. To address the limited knowledge available about the biological functions of milk traits, it is essential to consider TFs and their cooperation. To date, only a few studies have focused on the specific role of individual TFs within the MUG genome. In pursuit of this purpose, Yahyaoui et al. [37] conducted base excision sequence scanning and pinpointed an SNP in the promoter region of the beta-lactoglobulin gene linked to milk protein that did not affect the binding site of any TF in their study. Additionally, Zidi et al. [25] focused on scrutinizing the coding region of the sterol regulatory element-binding transcription factor 1, uncovering its connection with milk fat content and composition traits. More recently, Luigi-Sierra et al. [19] explored the genetic architecture of the udder in MUGs, detecting the presence of the *ATF3* gene in proximity to the SNP rs268273468, which was found to be correlated with the development of mammary gland tissue.

Previous studies on milk traits have primarily identified variants using conventional SNP-based analyses, focusing on additive effects and often overlooking the role of SNP–SNP interactions, or epistasis, in shaping these complex traits [26]. Furthermore, while transcriptional regulation plays a crucial role in quantitative traits, the specific functions of TFs within the MUG goat genome remain largely unexamined. To address these gaps, our study investigated the genetic architecture governing seven key milk traits in MUGs, using a genotype–phenotype dataset of 822 animals. Our approach integrated a two-step analysis pipeline: the first step used the random forest (RF) algorithm to evaluate the relative importance of each SNP in determining the traits of interest [38,39,40,41]; in the second step, we applied an information theory-based epistasis method, mutual information-based detection of epistatic SNP pairs (MIDESP) [27], to uncover complex genetic interactions that single-SNP analyses might overlook. Our analyses revealed distinct candidate genes associated with each trait, despite high phenotypic correlations among some traits. To further understand the regulatory mechanisms driving these gene–trait relationships, we employed the potentially collaborating transcription factor finder (PC-TraFF) algorithm [42,43], focusing on promoter regions to identify cooperative TFs specific to each trait. Although only a few genes are commonly associated with all traits, the PC-TraFF results suggest that a large number of TFs and their cooperative partners consistently participate in the regulatory networks underlying these milk traits in MUGs.

## 2. Materials and Methods

This section describes the genotype–phenotype dataset that was analyzed, as well as the research methodology.

### 2.1. Murciano-Granadina Goat Dataset

To elucidate the role of regulatory elements, such as TFs and their interactions, that could be essential in orchestrating the development of the seven milk traits, we analyzed a genotype–phenotype dataset previously set up by Guan et al. [24] to study the genetic basis of milk yield and composition in MUGs. The dataset contains the phenotypic data for 1023 MUGs, which includes dry matter percentage (DM), length of lactation/milk production days (DPM), fat percentage (FP), protein percentage (PP), lactose percentage (LP), milk yield at 210 days (MY), and somatic cell count (SCC). These traits were recorded across 15 dairy farms affiliated with CAPRIGRAN during the goats’ first lactation, where artificial insemination was the breeding method. While all the traits were standardized for a first lactation length of 210 days, DPM was an exception. A total of 822 goats were included in our analysis, after excluding those with insufficient or missing phenotypic data. Genotypic data for the animals were generated using the Goat SNP50 BeadChip, which contains 53,347 SNP markers, following the instructions provided by Illumina [44]. Following the criteria set out in [24], our analysis only included SNPs that (i) mapped to the autosomes; (ii) displayed a minor allele frequency of >0.01; (iii) did not deviate significantly from the Hardy–Weinberg expectation (*p*-value $>1\times 10^{-6}$); (iv) had genotype call rates >90 percent; and (v) had SNP call rates >90 percent. After filtering, we used 822 animals and 49,272 SNPs for our analyses.

### 2.2. Association Analysis

To identify SNPs potentially associated with the traits of interest in this study, we applied two distinct approaches: (i) a machine learning algorithm—specifically, RF—to assess the relative importance of each SNP (attribute) in predicting response variables (phenotype values); and (ii) an information theory-based methodology, the MIDESP algorithm [27], to detect epistatic interactions between pairs of SNPs and their corresponding phenotype values. In line with previous studies [45,46], we considered genes located within a 25 kb range around the identified SNPs as potential candidates for further analysis.

#### 2.2.1. Association Analysis Using Random Forest

In assessing the importance of SNPs for the prediction of the phenotype of interest, we applied an association analysis method anchored in RF-based feature selection, as described in previous studies [38,39,40,41]. Specifically, we used the Boruta algorithm [47], a tool tailored for RF-based feature selection, to calculate the importance scores of each SNP. These scores were instrumental in determining the relevance of the SNPs, to subsequently facilitate their ranking in phenotype prediction.

**Figure 1:**
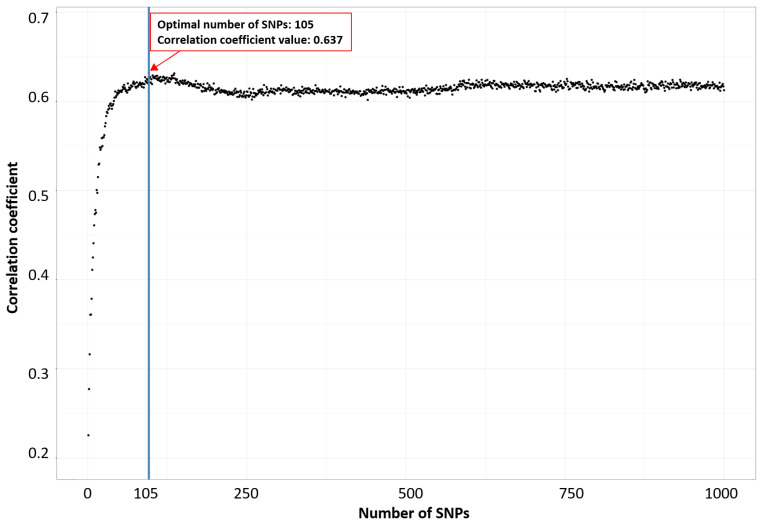
Identification of the optimal number of SNPs associated with milk yield at 210 days through using incremental feature selection.

Secondly, we utilized the incremental feature selection (IFS) technique, which prioritized SNPs based on their rankings in an RF model. This approach was used to identify the optimal number of features (SNPs) associated with a particular phenotype, as shown in Figure 1 for milk yield at 210 days, following the methodology suggested in [41,48,49,50]. During the application of IFS, SNPs were progressively added in descending order of their ranks within the feature set. Subsequently, an RF classifier was built, and its predictive performance was assessed based on the correlation coefficient. Supplementary Figures S1 and S2 provide an extended view of Figure 1 with 3000 and 5000 SNPs, enabling a comparative analysis of the impact of different SNP numbers.

#### 2.2.2. Epistasis Analysis Using MIDESP Algorithm

While single-SNP-based association analysis offers valuable insights into genotype–phenotype associations, it is now widely recognized that many complex traits arise from the interactions of multiple factors [27,51,52,53]. Therefore, the incorporation of epistasis could yield a more comprehensive understanding of these interactions by providing novel insights into the genetic architecture of complex traits that might be overlooked in a standard single-SNP analysis. To elucidate further the biological processes underlying milk traits in MUGs, we incorporated the MIDESP algorithm [27] in this study. Central to the MIDESP algorithm is the use of the mutual information (MI) metric, a powerful measure for assessing the strength of association between two or more variables. By considering both the marginal and joint probability distribution of the variables, the MI metric can account for linear as well as non-linear dependencies. Leveraging the MI metric, we applied the MIDESP algorithm to identify pairwise epistatic interactions between SNPs and the milk traits being analyzed. Consequently, the IFS technique was applied to identify the optimal number of SNP pairs associated with the phenotype of interest.

### 2.3. Identification of Transcription Factor Cooperation

In this study, we utilized the PC-TraFF algorithm [42] to identify cooperation between TF pairs by focusing mainly on the co-occurrence of their TF binding sites (TFBSs) in the promoter sequences of the candidate genes found for each trait. We let TFBS_A_ and TFBS_B_, etc., be the binding sites of TFs TF_A_ and TF_B_, respectively. The PC-TraFF algorithm computed the pointwise mutual information (PMI) score in six steps, using the joint probability $p({\mathrm{TFBS}}_{A},{\mathrm{TFBS}}_{B})$ and the marginal probabilities $p({\mathrm{TFBS}}_{A})$ and $p({\mathrm{TFBS}}_{B})$, to yield a PMI(TF_A_; TF_B_) value for each TF pair TF_A_ and TF_B_. In the final phase, the algorithm transformed the PMI(TF_A_; TF_B_) values into *z*-scores. The cooperation between TFs TF_A_ and TF_B_ was considered to be significant if the *z*-score was ≥3. The application of the PC-TraFF algorithm required pre-defined distance preferences of the TFBSs, position weight matrices (PWMs), and promoter sequences as input parameters. We implemented the algorithm with the recommended distance values, setting the minimum distance to ≥5 and the maximum distance to ≤20. For identifying putative TFBSs, we first extracted the promoter sequences (ranging from −500 bp to +100 bp relative to the transcription start site) of the candidate genes, using gene annotation and the ARS1 genome assembly of goats from the Ensembl database [54]. We then employed a non-redundant vertebrate PWM library from the TRANSFAC database [55], along with the Match [56] program, to scan these sequences for predicting the TFBSs.

## 3. Results

In this study, we analyzed a genotype–phenotype dataset of 822 MUGs to identify candidate genes and their regulatory mechanisms associated with seven milk-related traits. To achieve this, an RF-based feature selection algorithm in conjunction with the MIDESP approach was used to identify candidate SNPs, SNP pairs, and their corresponding genes for each trait (Table 2). The complete list of identified SNPs, SNP pairs, their corresponding genes, and gene pairs are listed in Supplementary Tables S1 and S2.

Although both methods aim to reveal important associations and their significance within the genotype–phenotype dataset, Table 2 shows that each method identified a considerably different number of associated SNPs and genes. The disparity can be attributed to the difference between their algorithms. Specifically, the RF-based feature selection method focuses primarily on assessing the importance of individual SNPs, whereas the MIDESP algorithm performs a stringent association analysis between SNP pairs and the traits of interest. The MIDESP algorithm also includes an additional filtering step that eliminates expected background associations, resulting in a dramatic reduction in the number of significant pairs identified. A closer examination of the identified candidate gene lists highlights the complementary nature of both association analysis methods, as they provide different types of information, resulting in a small overlap between the gene lists. Moreover, given the quantitative nature of the seven milk-related traits under study, it is likely that multiple genes with both additive and epistatic effects influenced them. Therefore, an integrated consideration of the candidate genes obtained from both analyses for each trait could be quintessential for understanding the genetic mechanisms controlling milk-related traits in MUGs.

**Figure 2:**
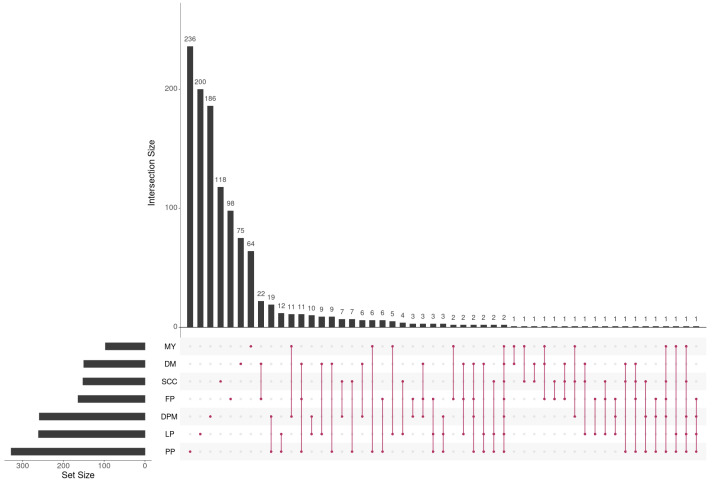
The number of genes and their overlap across the seven dairy traits represented in matrix layouts using the UpSet technique [58]. The maroon circles in the matrix layout are related to the traits that are part of the intersection. For the sake of clarity, not all intersections are displayed.

Figure 2 shows that the number of candidate genes, as well as the overlap between them, varied considerably across different traits. Interestingly, only two genes linked to *U6* and *5S-rRNA* were identified as common across all dairy traits, as depicted in Figure 2. It is well known that *U6* and *5S-rRNA* play crucial roles in regulating gene expression and protein synthesis as part of the ribosomal RNA family. Their presence suggests potential involvement in the regulation of milk production and composition, which is consistent with prior research highlighting their impact on milk traits [57].

Following the association analysis, we compiled a set of candidate genes for each trait and, subsequently, we extracted their promoter sequences. This enabled us to identify cooperative TFs regulating the genes that are associated with a particular trait.

### Identification of Cooperative Transcription Factors Regulating Seven Milk-Related Traits

**Figure 3:**
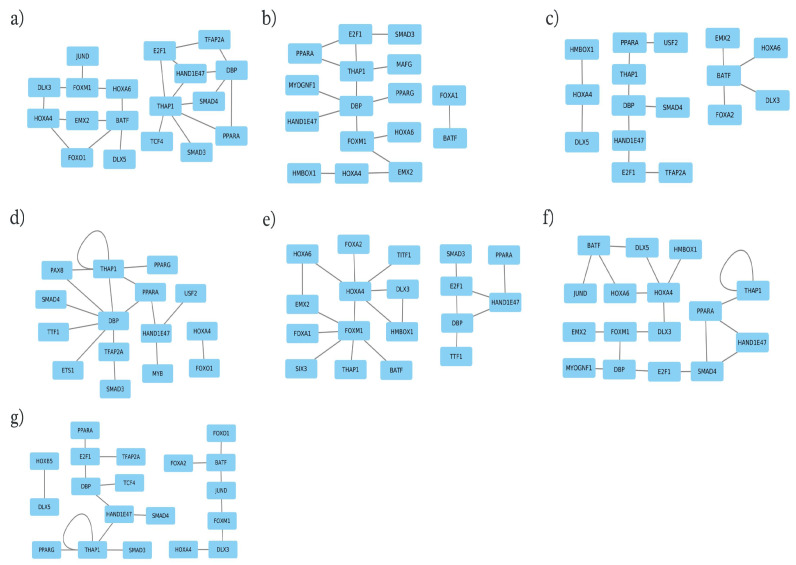
Cooperation networks for the transcription factor pairs of: (**a**) dry matter percentage; (**b**) milk production days; (**c**) fat percentage; (**d**) lactose percentage; (**e**) milk yield at 210 days; (**f**) protein percentage, and (**g**) somatic cell count traits.

Applying the PC-TraFF algorithm [42,43] to promoter sequences of candidate genes revealed significant cooperative TF pairs for each trait. To further elucidate the gene regulatory mechanisms of the underlying biological processes for the seven milk-related traits in the MUGs, we extended our analysis by constructing trait-specific cooperation networks based on the identified TF pairs, as described in our previous studies [43,59,60,61,62]. As illustrated in Figure 3, the nodes and edges in these networks represented the TFs and their cooperative interactions, respectively. A closer look at these networks reveals that they comprised only 32 unique TFs participating in the formation of 118 TF pairs, which suggests that TFs switch partners according to their functional roles in different traits [60,61]. Additionally, to highlight the activity of these TFs during the lactation period, their gene expression levels, similar to those reported by Zeidler et al. [62], are provided in Supplementary File S1, Table S3 and Figure S3.

The number of individual TFs and TF pairs is given in Table 3 for each trait. Remarkably, the TFs DBP, HAND1E47, HOXA4, PPARA, and THAP1 were consistently identified across all milk traits. This overlap of TFs in cooperation networks for all milk traits suggests common regulatory processes in mammary gland tissue.

The D-site binding protein (DBP) is a member of the proline and acidic amino acid-rich basic leucine zipper (PAR bZip) TF family, which includes other members, such as the hepatic leukemia factor and the thyrotroph embryonic factor [63,64]. DBP plays a pivotal role in regulating the expression of clock genes, which are essential for controlling circadian rhythms [65] and influence the activity of various enzymes [66]. Circadian rhythms, representing adaptations to daily environmental variations, are under the regulatory control of PAR bZip factors [63]. Consequently, this circadian system governs metabolic processes and physiological activities, which are crucial determinants of milk quality and quantity in mammals [67,68].

The basic helix–loop–helix TF family member, HAND1E47, plays crucial roles in the development of the mammary gland by regulating essential processes, such as cell proliferation, differentiation, and apoptosis [69,70]. Furthermore, Kumar et al. [71] revealed the participation of HAND1E47 in the promoter region of the *S100A8* gene, elucidating its role in the immune function within mammary glands. This is further substantiated by the expression of *S100A8* in milk somatic cells, particularly following infections in goat mammary glands [72]. Another interesting factor is HOXA4, a member of the homeobox (HOX) protein family characterized by its DNA-binding homeobox domain, which plays a crucial role in regulating early embryo development patterns and subsequent developmental processes [73,74]. HOXA4 plays a role in various physiological and pathological processes in organisms, mediated by well-established signaling pathways [73,75,76,77]. It is also involved in processes like cell proliferation, differentiation, apoptosis, signal transduction, and tumor suppression [78,79]. Furthermore, its regulatory role has been observed in controlling milk traits, aligning with the findings of Pellacani et al. [80], who identified this factor as an active enhancer for multiple genes in mammary gland epithelial cells. In our analysis, we also identified the PPARA factor, a member of the peroxisome proliferator-activated receptors (PPARs), which are well-recognized for their crucial role in governing lipid metabolism [81] and regulating cell proliferation and the cell cycle [82]. Moreover, PPARA has been identified as a key regulator of activities in both epithelial and stromal cells within mammary glands [83], influencing the synthesis of milk fat content.

Furthermore, we identified THAP1, a member of the Thanatos-associated proteins (THAP) family. THAP1 features a simplified proline-rich region, a coiled-coil domain, and a binary nuclear localization sequence [84]. It is involved in cell division, apoptosis, and transcription control [85,86], and it is broadly expressed in various tissues, including the brain, as well as extra-neural tissues such as the thyroid gland, prostate, liver, kidney, and blood [86,87]. Mutations in THAP1 can result in loss of function, leading to transcription dysregulation and dystonia-like conditions in humans [85]. Interestingly, THAP1 is also implicated in clock-controlled gene regulation [60,88], affecting physiological processes that influence mammary gland growth and lactation competence [89].

A closer look at the cooperation networks in Figure 3 further reveals that they frequently contain members of the DLX, FOX, HOX, PPAR, and SMAD families. In particular, the members of the distal-less homeobox (DLX) family play a vital role in embryonic ectoderm development and the formation of structures such as teeth, hair follicles, and mammary glands [90]. Exhibiting dual functionalities, they can act as either activators or repressors, depending on their partner choice [91]. Additionally, DLX members have been studied for their role in regulating mammary branching morphogenesis [92], as well as for their involvement in the immune response in the udder [93]. Interestingly, our analysis revealed cooperation between the DLX and HOX family members (e.g., the DLX3–HOXA4 pair for DM, MY, PP, and SCC, and DLX5–HOXA4 cooperation for FP and PP traits) that are essential for governing both body segmentation and developmental processes [94,95,96].

In this study, we identified several forkhead box (FOX) family members (FOXA1, FOXA2, FOXM1, and FOXO1), which are known to play pivotal roles in various aspects of mammalian development and disease, including immunological regulation, glucose and lipid metabolism, and the biological aging process [97,98]. Specifically, FOXA1, FOXM1, and FOXO1 have been recognized as key regulators in the development of the mammary glands and modulating lactation hormones during the reproductive cycle and pregnancy in mammals [80,99,100,101].

Furthermore, our study revealed a notable cooperation between the TFs FOXA1 and FOXM1 concerning the MY trait, which is pivotal for regulating hormones [102] that may significantly influence both milk production and immunity.

Other interesting TFs in our analysis included HOXA6 and HOXB5, which are members of the HOX family. These TFs govern cell differentiation, cellular functions, cell proliferation, embryonic development, and tissue stability by regulating the promoter regions of a diverse array of target genes [79]. In mammals, HOX genes play a critical role in shaping body anatomy, the skeletal and central nervous systems, and regulating mammary glands and lactation [103]. Additionally, they relate to genetic susceptibility to mammary gland tumors [79] and to human breast cancer [104].

Similarly, we also identified another member of the PPARs family that plays a vital role as a nuclear receptor crucial for processes such as lipid metabolism and immune responses [105]. Within the context of mammary glands, they hold a pivotal role in the regulation of milk fat synthesis, secretion, and transportation [106]. Notably, as elucidated by Liu et al. [107], PPARG is expressed not only within the nucleus of bovine mammary epithelial cells but also upregulates the expression of key genes, such as fatty acid synthase (*FASN*) and acyl-CoA synthetase short-chain family member 2 (*ACSS2*) during the lactation phase in goat mammary gland epithelial cells [108]. Collectively, these studies reveal the positive regulatory role of the PPARs family members in the synthesis of milk fat in ruminants [106].

Suppressor of mothers against decapentaplegic (SMAD) family members SMAD3 and SMAD4, on the other hand, modulate transcriptional activities either independently or in combination with various co-regulators [109]. Their scope includes tissue development and homeostasis, pivotal for cellular fate in multicellular systems [110]. They also interface with the transforming growth factor beta (TGFβ) signaling pathway [111], although TGFβ shows a negative correlation with certain milk components like casein production and milk secretion [112].

Altogether, our findings on TFs provide critical insights into the regulatory mechanisms of candidate genes, which may help decipher the genetic programs governing specific biological processes related to the development of various milk traits in mammary gland tissue in MUGs. Particularly, milk synthesis results from complex interactions among several tissues and organs, including the immune system, adipose tissue, liver, muscles, and, most importantly, the mammary gland tissue [113], where TFs play a key role in regulating gene expression leading to the production of lactose, protein, fat, and other milk components. This process highly influences both the production and quality of milk. Consequently, the elucidation of TFs and their trait-specific partner choice, on the one hand, enriches our understanding regarding individual milk traits, and, on the other hand, could lead the way for future research to further decipher the genetic basis of these traits and their practical application in MUG breeding.

## 4. Discussion

Goat milk is consumed as a beverage globally and is a key ingredient in cheese production. The quality of cheese produced from goat milk depends on its fat and protein composition, which are known to be inversely related to milk yield [4,114]. In contrast, the MUG breed demonstrates a significant positive correlation between milk yield and its fat and protein content, along with low SCC, resulting in superior milk quality [4,114]. This breed also maintains consistent milk production during reproductive seasonality [1]. Recent studies [6,17,18,24] have actively explored the genetic and genomic basis of these distinct milk traits in MUGs, aiming to understand the inherent differences and to develop precise breeding strategies for sustainable dairy production. This focus on precision breeding and optimizing dairy production underscores the importance of genetic and genomic research in this field.

In this context, Guan et al. [24] conducted an association analysis using a linear mixed model to explore the relationship between SNP genotypes and dairy traits, identifying, in total, 26 quantitative trait loci linked to seven milk traits (DM, DPM, FP, LP, MY, PP, and SCC) in MUGs. While single-SNP analysis methods, like those used by Guan et al. [24], are effective in identifying key SNPs, they capture only a portion of the genetic variation and make a modest contribution to the variance of complex traits [115,116]. Given the polygenic nature of quantitative traits and the potential for epistatic interactions, focusing solely on individual SNP effects may overlook the broader genetic architecture of these traits. To address this, we employed two distinct approaches in our study: the machine learning-based RF and the information theory-based MIDESP algorithms. These methods were used to identify genotype–phenotype associations, accounting for both single-SNP associations and epistatic interactions. Although both methods aimed to unravel the complex genetic underpinnings of the seven milk traits in the MUGs, Table 2 demonstrates that they identified considerably different candidate genes for each trait, using the same dataset. A limited number of common genes were found for each of the seven milk traits, using both methods. To gain a more comprehensive understanding of the genomic architecture of these traits, it was crucial to consider the combined set of candidate genes identified by both the RF and MIDESP algorithms. This integrative approach has the potential to uncover genetic patterns with subtle individual SNP effects and their significant interactions, revealing the intricate genetic basis of these traits. Of particular interest, we further made a pairwise comparison using combined gene sets among the seven milk traits (Figure 2). Despite the known associations between mammary gland tissue and these traits, our comparative analysis revealed a striking pattern of disparity in the underlying genes for these traits, suggesting a more complex genetic interplay that requires further investigation.

Hence, another fundamental step in our study was the application of the PC-TraFF algorithm [42] for analyzing TFs and their cooperation, since TFs play a pivotal role in regulating gene expression, thereby strongly influencing the traits by directing biological processes and developmental pathways [117,118,119]. These are crucial for understanding the complex genetic programs and the transcriptional machinery of these traits. It is widely recognized today that TFs change their partners depending on their biological functions, and their cooperation is essential for the precise orchestration of specific genetic programs [61]. Previous studies have demonstrated that the preferential partner choice of TFs is achieved through a non-random process, which is strongly influenced by their specific roles in distinct biological functions and the cellular context [59,60,61,118,120]. In particular, within this context, we identified the TF DBP as a central hub for DPM and LP traits, consistently cooperating with TFs HAND1E47, SMAD4, E2F1, and THAP1 across various traits (Figure 3). The role of the DBP factor in immunity and milk production in sheep and cattle [121,122] has also been discovered, thereby strengthening our results. Similarly, Lemay et al. [68] uncovered the role of DBP in regulating mammary development by controlling circadian rhythms during lactation in mice. In contrast, Casey et al. [123] noted DBP’s downregulation for intracellular circadian rhythms in mammary gland tissue during the pregnancy-to-lactation transition. This difference in the role of DBP in the mammary gland tissue may be attributed to the transition from one phase of pregnancy to the next productive phase of lactation. Additionally, we frequently observed the cooperation of DBP with HAND1E47, possibly suggesting their potential involvement in regulating processes related to immunity and milk yield synthesis in the udder in response to circadian rhythms during the lactation period, as explained in Section 3.

Similarly, SMAD3/4 were involved in regulating various milk traits in our study. They influence lactation performance through signaling pathways within the mammary gland, as found in mammals [124]. Our findings revealed the cooperation of E2F1 with SMAD3/4, particularly concerning protein percentage, dry matter percentage, and milk yield. This collaboration was also identified in mammary cancer, where it acts as a suppressor [125,126], and both factors are additionally implicated in the regulation of fat metabolism [127].

Another important TF identified in our findings, BATF, serves as a central node in regulating fat percentage along with other milk production traits. The role of BATF in immunity and fat metabolism, as identified in previous studies [128,129], aligns with our results. Remarkably, the cooperation between FOXO1 and BATF, identified for the DM trait, has a substantial impact on correct B cell proliferation [130]. Additionally, BATF and FOXA1 factors have been previously noted in various processes, including T cell development [131] and genomic regulatory regions in mammary glands epithelial cells [80]. Additionally, our findings indicate THAP1’s frequent cooperation with PPARA for different traits, suggesting its regulatory role in gene expression within the mammary glands. This contributes to lactation by assisting in the synthesis of milk constituents. Likewise, the positive regulatory role of PPARA in goat mammary glands epithelial cells for the synthesis and transportation of fat has been confirmed by various studies [106,132].

Overall, our findings revealed a notable divergence in the candidate genes among the seven milk traits in the MUG breed, while highlighting a surprising similarity in the TFs regulating them. This observation underscores a fundamental aspect of gene regulation: distinct gene profiles are governed by specific sets of TFs. Our analysis further unveiled a critical mechanism in the regulation of specific traits—most TFs are shared by all networks (Figure 3), yet they exhibit dynamic partner-switching behavior. This could suggest a highly evolved regulatory flexibility, wherein TFs modulate different phenotypic traits by changing their cooperation partners. These insights not only deepen our understanding of gene regulatory networks but also pave the way for further investigation into the intricate role of TFs in phenotype determination. These factors play direct or indirect roles in lactation through the synthesis of milk components. Specific regulatory factors such as FOXA1, FOXM1, BATF, DBP, and HAND1E47 are involved in regulating target genes crucial for both milk production and udder immunity, thereby influencing milk quality. However, it is crucial to note that our knowledge about the effects of these regulators is still evolving.

While this study focused on TFs and their preferred partner choices influencing milk traits in the MUG breed, similar regulatory mechanisms may be relevant across other dairy breeds, such as sheep. Many TFs involved in milk production might be conserved among ruminants [106], suggesting that consideration of TF cooperation could potentially be used for improved dairy performance. However, breed-specific factors, such as unique genetic backgrounds and environmental adaptations, could influence the extent and nature of TF interactions. Further studies across diverse dairy breeds would help validate the broader applicability of these regulatory mechanisms.

## 5. Conclusions

Milk production is a primary trait of the MUG breed, and understanding the genetic framework behind complex milk traits, which are regulated by multiple genes, is essential for optimizing milk yield and quality. In this study, we applied a machine learning approach and an information theory-based approach to analyze genotype–phenotype data. Our results reveal that, although distinct sets of genes contribute to different milk traits, their regulatory factors show strong similarities. Specifically, networks of TF pairs indicate that five key families dominate, comprising 13 of the 32 unique TFs identified. This underscores the importance of examining the cooperative interactions and partner-switching behavior of these TFs, based on their roles in regulating various traits, to gain valuable insights into the biological mechanisms underlying milk production in MUGs.

## Figures and Tables

**Table 1:** Representative studies on association analysis of milk traits in MUG goats.

Authors	Synopsis of Study	Type of Data
Badaoui et al. [14]	Assess the association of Acetyl-coenzyme A carboxylases α genotypes with milk traits by determining the polymorphisms.	Genotype–phenotype
Badaoui et al. [15]	Study of the genetic variants affecting milk quality and cheese manufacturing components.	Genotype–phenotype
Zidi et al. [16]	Identification of SNPs that create or modify microRNA target sites in caprine casein genes.	Genotype–phenotype
Zidi et al. [22]	Examine the association between the coding region of the caprine malic enzyme 1 (*ME1*) gene and the composition of milk fatty acids.	Genotype–phenotype
Zidi et al. [23]	Analysis of the association between the goat stearoyl-CoA desaturase 1 (*SCD1*) gene and milk quality traits.	Genotype–phenotype
Manunza et al. [25]	Exploration of the genetic variability in the caprine *SREBF1* coding region and its association with milk composition and fat contents in dairy goats.	Genotype–phenotype
Guan et al. [6]	Exploration of the genetic signatures generated by artificial selection for dairy and pigmentation traits in MUGs.	Genotype–phenotype
Guan et al. [24]	Analysis of the molecular basis of lactation as well as pinpointing the genetic variables that influence the quantity and quality of milk.	RNA-seq and genotype–phenotype
Inostroza et al. [17]	Identification of casein complex haplotype variants in MUGs and their effects on milk traits.	Genotype–phenotype
Inostroza et al. [18]	Assessment of the dominant and additive effects of casein complex SNPs, as well as their epistatic interactions, on genetic parameters, breeding values, and milk traits in MUGs.	Genotype–phenotype
Luigi-Sierra et al. [19]	Genomic mapping of body, udder, and leg conformation traits in MUGs.	Genotype–phenotype
Luigi-Sierra et al. [20]	Determination of inbreeding levels in a MUG resource population using different genomic coefficients to quantify the influence of inbreeding depression on dairy phenotypes.	Genotype–phenotype
Luigi-Sierra et al. [21]	Identification of the genetic regions associated with many morphological traits using a GWAS in MUGs.	Genotype–phenotype

**Table 2:** Results of single-SNP-based GWAS and SNP–SNP interaction epistasis-based GWAS.

Traits	RF-Based Feature Selection		Epistatis Analysis by MIDESP		Common Genes
	SNPs	Genes	SNP Pairs	Genes	
DM	580	120	122	38	8
DPM	550	124	674	164	29
FP	604	105	217	72	13
LP	369	91	789	187	17
MY	105	22	191	78	06
PP	243	56	991	291	19
SCC	363	74	377	96	18

**Table 3:** Significant TFs, cooperative TF pairs, and the top-three pairs for each trait, as identified by PC-TraFF. The pairs are sorted in ascending order, based on their *z*-scores provided by PC-TraFF.

	Number of		
Trait	TFs	TF Pairs	Top-Three TF Pairs
DM	18	22	[PPARA–THAP1]; [HAND1E47–DBP]; [E2F1–TFAP2A]
DPM	16	15	[DBP–THAP1]; [HAND1E47–DBP]; [PPARA–E2F1]
FP	16	13	[HAND1E47–DBP]; [DBP–SMAD4]; [HOXA6–BATF]
LP	15	15	[THAP1–THAP1]; [DBP–THAP1]; [DBP–SMAD4]
MY	18	19	[HAND1E47–DBP]; [HOXA4–HMBOX1]; [FOXM1–BATF]
PP	16	17	[THAP1–THAP1]; [DBP–FOXM1]; [PPARA–THAP1]
SSC	19	16	[HAND1E47–DBP]; [THAP1–THAP1]; [PPARA–E2F1]

## Data Availability

No new data were created or analyzed in this study. Data sharing is not applicable to this article.

## Supplementary Materials

The following supporting information can be downloaded at https://www.mdpi.com/article/10.3390/biology13110929/s1: Figure S1: Identification of the optimal number of SNPs associated with milk yield at 210 days through using incremental feature selection by considering 3000 SNPs.; Figure S2: Identification of the optimal number of SNPs associated with milk yield at 210 days through using incremental feature selection by considering 5000 SNPs; Figure S3: Expression values of TF genes (DBP, HAND1E47, HOXA4, PPARA, and THAP1) in mammary gland tissue of Murciano-Granadina goats at three different time points; Table S1: Number of SNPs and the corresponding genes identified using RF-based GWAS for each trait; Table S2: Number of SNP pairs and the corresponding genes identified in SNP–SNP interaction epistasis analysis for each trait; Table S3: Expression values of TF genes, measured in three different time points. The expression values are given as counts per million (CPM) values.

## Abbreviations

The following abbreviations are used in this manuscript:

MUG	Murciano-Granadina goat
CAPRIGRAN	National Association of Murciano-Granadina Goat Breeders
SNPs	single-nucleotide polymorphisms
TFs	transcription factors
TFBSs	transcription factor binding sites
MIDESP	mutual information-based detection of epistatic SNP pairs
RF	random forest
PC-TraFF	potentially collaborating transcription factor finder
DM	dry matter percentage
DPM	milk production days
FP	fat percentage
PP	protein percentage
LP	lactose percentage
MY	milk yield at 210 days
SCC	somatic cell count
IFS	incremental feature selection
MI	mutual information
PMI	pointwise mutual information
PWM	position weight matrices
DBP	D-site binding protein
HOX	homeobox
PPARs	peroxisome proliferator activated receptors
THAP	Thanatos-associated proteins
DLX	distal-less homeobox
FOX	forkhead box
SMAD	suppressor of mothers against decapentaplegic
