# Supplementary material for: Computational Identification of Milk Trait Regulation Through Transcription Factor Cooperation in Murciano-Granadina Goats
Source: Biology (Basel). 2024 Nov 15;13(11):929. doi: 10.3390/biology13110929 (PMC11591944; doi:10.3390/biology13110929)
Supplement: Supplementary file 1 [file biology-13-00929-s001.zip › Supplemnetray File S1.pdf]

Expression of transcription factor genes

Gene expression of the identified transcription factors of our analysis is adopted from Guan et al. (https://jasbsci.biomedcentral.com/articles/10.1186/s40104-020-00435-4). Data was sampled from mammary glands at 78 d (T1, early lactation), 216 d (T2, late lactation), and 285 d (T3, dry period) post-parturition and mRNA profiles of seven Murciano-Granadina goats were created.

Table S3: Expression values of TF genes, measured in three different time points. The expression values are given as counts per million (CPM) values.

Name	T1	T2	T3
HAND1/E47	7.7	15.99	15.75
HMBOX1	28260.94	17236.55	21706.95
DLX5	1670.1	1135.25	996.16
JUND	190245.67	232853.39	148196.27
BATF	1062.09	1231.18	2795.56
MYB	3178.59	3901.41	12875.33
SIX3	261.68	255.83	303.18
DLX3	2732.2	3741.52	7087.34
THAP1	8496.75	9002.02	8748.93
SMAD3	26252.19	27117.99	42598.85
FOXA2	0	63.96	98.44
USF2	95480.71	99565.89	91092
HOXA6	1770.16	1798.81	2122.26
MAFG	8065.76	8370.44	8772.55
E2F1	1454.61	2622.26	2649.88
ETS1	42599.21	46281.27	60770
PPARG	12006.28	12111.96	11930.36
TTF1	23827.85	26526.39	31971.78
MYOGNF1	19040.73	20370.47	20175.29
FOXO1	142920.91	124205.53	110767.24
TFAP2A	120986.36	114819.76	140715.19
SMAD4	83620.66	69218.04	80449.18
TCF4	57160.67	56562.44	78043.42
HOXA4	2955.39	3813.47	3732.67
PAX8	1115.97	1279.15	1917.52
DBP	74146.48	66867.6	60998.37
HOXB5	446.39	383.75	531.55
FOXA1	18309.58	24503.73	34078.29
EMX2	338.64	703.53	818.98
FOXM1	977.43	1566.96	1937.21
PPARA	30608.32	21873.48	11103.5

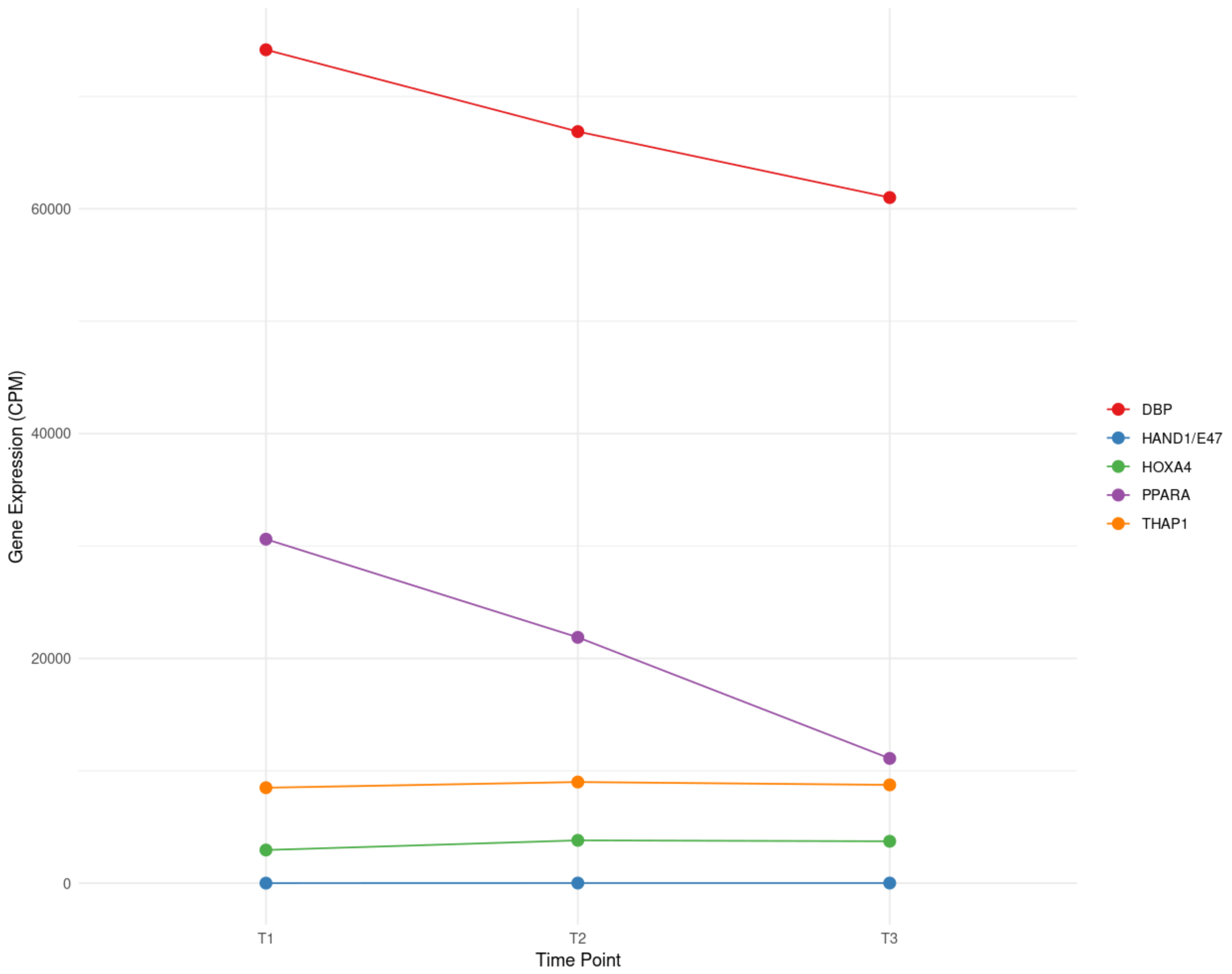

Figure S3: Expression values of TF genes (DBP, HAND1E47, HOXA4, PPARA, and THAP1) in mammary gland tissue of Murciano-Granadina goats at three different time points.
