# Supplementary figures and images for: Computational Identification of Milk Trait Regulation Through Transcription Factor Cooperation in Murciano-Granadina Goats
Source: Biology (Basel). 2024 Nov 15;13(11):929. doi: 10.3390/biology13110929 (PMC11591944; doi:10.3390/biology13110929)

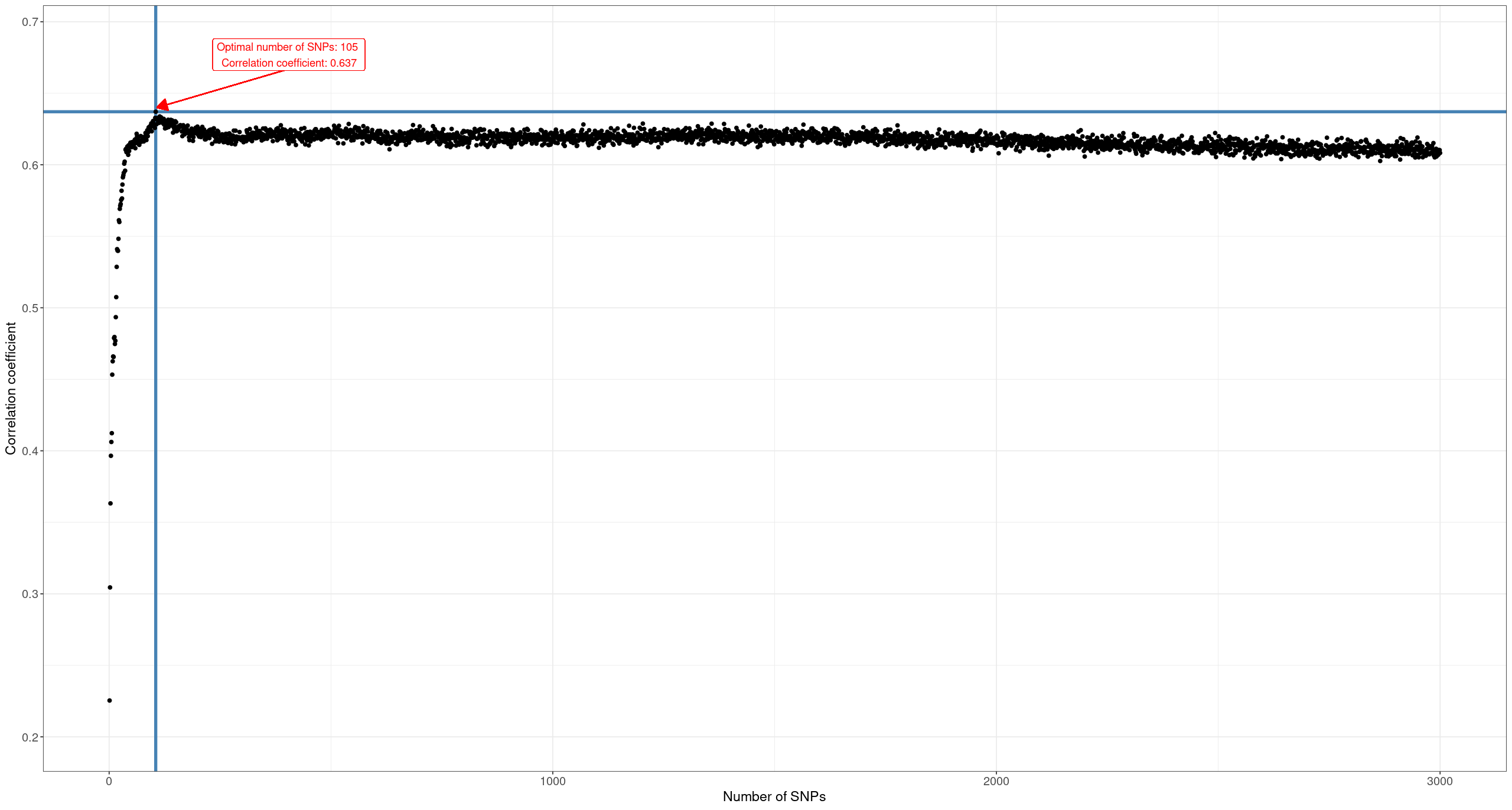

Supplement: Supplementary file 1 [file biology-13-00929-s001.zip › Supplemnetray Figure_S1.png]

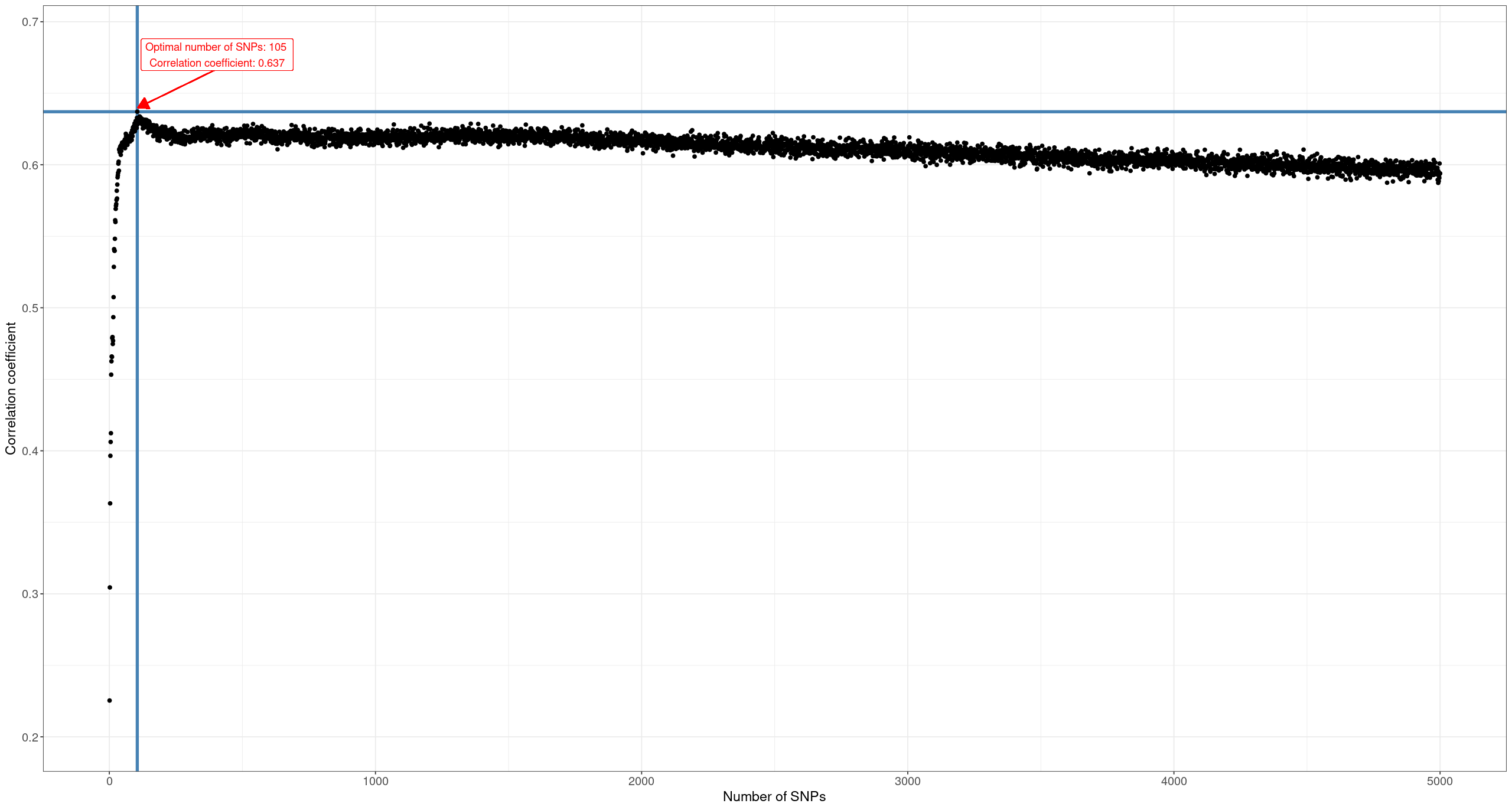

Supplement: Supplementary file 1 [file biology-13-00929-s001.zip › Supplemnetray Figure_S2.png]
